# The effect of multiple allergen immunotherapy on exhaled nitric oxide in adults with allergic rhinitis

**DOI:** 10.1186/1710-1492-9-31

**Published:** 2013-08-19

**Authors:** Michele Columbo, Bruce Wong, Reynold A Panettieri, Albert S Rohr

**Affiliations:** 1 Asthma, Allergy and Immunology Specialists, 875 County Line Road-Suite 107, Bryn Mawr, PA 19010, USA; 2 Center for Clinical Epidemiology and Biostatistics, University of Pennsylvania School of Medicine, Philadelphia, PA, USA; 3 Pulmonary, Allergy and Critical Care Division, University of Pennsylvania School of Medicine, Philadelphia, PA, USA

**Keywords:** Allergen immunotherapy, Nitric oxide, Allergic rhinitis

## Abstract

**Background:**

There is a lack of objective measures of the clinical efficacy of allergen immunotherapy which relies on patients’ perception about the effect of this treatment. We studied whether the fraction of exhaled nitric oxide is affected by multiple allergen immunotherapy in polysensitized adult subjects with allergic rhinitis. We also looked for associations between exhaled nitric oxide and subjects’ demographics, symptom scores, and pulmonary function tests.

**Methods:**

Twenty adult, polysensitized subjects with seasonal and perennial allergic rhinitis who chose to undergo allergen immunotherapy were enrolled. They were evaluated at baseline, and 4, 8, 12, 24, and 52 weeks later. Exhaled nitric oxide was reported as the mean of triplicate determinations.

**Findings:**

Our results indicate that multiple allergen immunotherapy did not affect exhaled nitric oxide levels and such levels did not correlate with subjects’ demographics and pulmonary function tests. However, exhaled nitric oxide was associated with rhinoconjuctivitis and asthma symptom scores at the end of the study.

**Conclusions:**

In polysensitized adult subjects with allergic rhinitis, exhaled nitric oxide levels are unaffected by multiple allergen immunotherapy.

## Findings

### Background

The fraction of exhaled nitric oxide (FENO) is elevated in subjects with allergic rhinitis compared to healthy controls, although less than in asthmatics [1]. Increased FENO levels have been proposed as indicative of eosinophilic airway inflammation and FENO is typically reduced by steroids, drugs known to decrease such inflammation [2]. It could be hypothesized that FENO levels could be used as an objective measure of the severity of allergic rhinitis.

Treatment of asthmatic children and adults with a monoclonal humanized anti-IgE antibody, omalizumab (Xolair) decreased FENO levels [3,4] and elevated FENO levels can help predicting the response to this treatment [5]. In contrast, conventional single allergen immunotherapy (IT) in mostly polysensitized asthmatic children and adults did not significantly affect FENO [6,7]. To our knowledge, there is no information concerning the effect of multiple allergen IT on FENO. Also as there is a lack of objective parameters to assess the efficacy of IT, we postulated that multiple allergen IT may modulate FENO levels in adult patients. Multiple allergen IT is routinely employed in the USA to treat polysensitized individuals with allergic rhinitis and asthma. FENO is positively associated with the number of positive skin tests in children with asthma and allergic rhinitis [8]. We examined associations among FENO and subjects’ demographics and the number of positive skin tests. As subjects with asthma were allowed to participate, we addressed associations among FENO and Asthma Control Test (ACT) scores and spirometric measurements.

### Methods

Twenty adult subjects just diagnosed with allergic rhinitis who elected to begin IT in an Allergy and Immunology practice in suburban Philadelphia were recruited and enrolled in the study. Prick tests were performed directly before study entry using a panel of 38 indoor and outdoor allergens (Greer, Lenoir, NC). Current or past smokers were excluded. This study was approved by the Main Line Hospitals Institutional Review Board and registered on ClinicalTrials.gov (NCT01318954).

The study subjects were followed for one year with evaluations including a brief history, physical exam, collection of ACT and reflective Allergic RhinoConjunctivitis Symptoms (ARCS) scores at baseline and at 4, 8, 12, 24 and 52 weeks. Allergic RhinoConjunctivitis Symptoms (ARCS) score is a scoring system for five groups of symptoms: 1) sneezing, 2) rhinorrhea (postnasal drip), 3) itchy nose/palate/throat, 4) itchy/watery or red eyes, and 5) nasal congestion. The study subjects were instructed to grade each of the five symptoms they were experiencing at the time of the visit on a scale including 0 (no symptom), 1 (mild), 2 (moderate), 3 (severe), 4 (very severe). The Asthma Control Test (ACT) is a commonly used tool that allows patients to grade their asthmatic symptoms. Patients report on a scale from 1 (severe) to 5 (no symptoms) their asthmatic symptoms in the previous four weeks by answering five questions about their asthma. Values over 19 are considered as indicative of good asthma control. Three subjects discontinued the IT within three months and were not included in the analysis. Seventeen subjects completed the study.

Allergy vaccines were prepared according to the subjects’ sensitivities, using purified extracts (Greer, Lenoir, NC; Hollister Stier, Spokane, WA; ALK Abello’, Port Washington, NY), and an initial dose equivalent to 1:500 of the maintenance dose. Such maintenance doses of the individual allergens were within the range of the published guidelines. The injections were given weekly while the vaccine’s dose was increased and administered every four weeks as maintenance. The IT was administered as two injections, containing perennial and pollen allergens, respectively. If scheduled for the same day, the allergy injections were administered after the study visit. All of the subjects who completed the study suffered from both perennial and seasonal allergic rhinitis. The most common sensitivities were dust mite (80%), ragweed (70%), and cat (65%). Subjects’ compliance with the IT schedule was excellent. In particular, >90% of the injections were given in seven days (± 2 days) during the dose escalation phase and in 28 days (± 7 days) during the maintenance phase of the IT.

Spirometric values were obtained according to the ATS/ERS guidelines by a KoKo Spirometer (nSpire Health, Inc, Longmont, Colorado). FENO was measured in triplicate determinations by NIOX MINO (Aerocrine, Morrisville, NC) according to the ATS/ERS guidelines and reported as ppb as the mean of the three determinations.

Statistical analysis was performed using STATA v10 (College Station, TX). Descriptive variables are expressed as means and standard deviation, Multiple linear regression was used to test association between groups of variables and group differences were tested using unpaired t-tests. Significance was accepted at alpha = .05 with no adjustment for multiple comparisons.

### Results

Table 1 summarizes the subjects’ characteristics at baseline. If only the subjects who completed the study were reported, the data were essentially unchanged. Eight subjects had mild intermittent and one had mild persistent asthma.

**Table 1:** Subjects’ Characteristics at Baseline

Female/Male	13/7
Age, yr (range)	36.3 ± 12.8 (21–71)
BMI (range)	25.5 ± 4.8 (20–38)
Inhaled Steroids	1/20
Nasal Steroids	6/20
Nasal Antihistamines	4/20
Oral Antihistamines	5/20
Number of positive allergy tests (range)	10.4 ± 7.3 (2–25)
Asthma	9/20
ARCS Score	6.3 ± 3.8
ACT Score	23 ± 4.1
FENO (ppb) (range)	32.4 ± 19.8 (8–88)
FEV_1_ (%)	101.7 ± 12.2
FEV_1_/FVC	0.79 ± 0.07
FEF_25-75%_	90.8 ± 24.2

Some results are expressed as the mean ± S.D. N = 20.

There were no significant differences between the values of FENO at consecutive study visits in all study subjects. Similarly, there was no significant difference in FENO values between the first and the last visits (33.5 ± 19.5 vs. 40.5 ± 27.5 ppb, respectively, p = 0.4). In the asthmatics, FENO showed a trend for an increase after IT (40.1 ± 19.9 ppb at baseline vs. 50.6 ± 29.9 ppb at the last visit, n = 9), but this was not statistically significant (p = 0.4). This was similar in the subjects without asthma (26 ± 17.1 vs. 28.9 ± 20.2, respectively, p = 0.76, n = 8). The reflective ARCS scores were only collected at the six study visits in subjects with multiple allergic sensitivities. The subjects’ starting date of IT was not synchronized with their pollen season. Therefore, such scores could not be used to assess the clinical efficacy of IT. However, they slightly increased up to the third visit (from 6.4 ± 4.1 to 7.4 ± 4.8) and then progressively decreased in the subsequent visits until the last study visit (to 4.9 ± 4). Whereas the ARCS scores did not vary significantly between first and last study visit (p = 0.16), they were lower at the last visit when compared to the third visit (p = 0.03). ACT scores and pulmonary function tests, both high at baseline, did not vary significantly throughout the study period and were similar in asthmatic and non-asthmatic subjects (data not shown). The effect of IT on the utilization of drugs could not be reliably assessed because of the low use at baseline.

We found no association between FENO and number of positive skin tests (p = 0.9), or age, sex, and Body Mass Index (BMI) (p > 0.14). FENO was higher in subjects with asthma (40.1 ± 19.9 vs. 26 ± 17.1 ppb), but this did not reach statistical significance (p = 0.09) likely due to the small number of observations. Similarly, we found no association among FENO and FEV_1_, FEV_1_/FVC, or FEF_25-75%_ at baseline and at the end of the study. At the final study visit, FENO was positively associated with ARCS scores and negatively correlated with ACT scores (p = 0.01 for both). Eighty eight percent of the subjects who completed the study judged the IT clinically effective. This conclusion was based on the subjective, yet significant reduction of their rhinoconjunctivitis symptoms over the year of treatment when compared to their symptoms’ severity in the previous years.

### Discussion

FENO was elevated (>30 ppb) in our study subjects but it was unaffected by multiple allergen IT. Similar results were obtained when only asthmatic subjects were included in the analysis.

Unlike studies in children, FENO was not associated with the number of positive skin tests. As in other studies [9], FENO was not associated with age, sex, or BMI in our study subjects. Although we did not find an association with asthma, it is likely that a larger number of observations would have resulted in a positive association.

Similar to other studies [10], including ours of FENO in elderly asthmatics [11], we found no association between FENO and FEV_1_, FEV_1_/FVC, or FEF_25-75%_ throughout the study period.

FENO was not associated with ARCS and ACT scores at baseline and throughout most of the study period. However, at the end of the study, we found a significant positive association between FENO and ARCS and a negative association between FENO and ACT scores. The reasons for these correlations are unclear. Such findings may have occurred by chance. However, we speculate that IT, while not leading to significant variations in FENO levels, may induce biologic changes that cause such associations.

Limitations of our study include the small sample size which limits the study’s ability to detect small changes in FENO if the FENO measurements are relatively unresponsive to change. The small sample size could have also prevented us from observing weaker associations between the different parameters. In addition, as it is routinely done in clinical practice, the efficacy of IT was subjectively established by the study participants.

### Conclusion

In conclusion, in our pilot study FENO levels were unaffected in subjects undergoing multiple allergen IT. The results of our study will require confirmation in larger subject cohorts.

## Abbreviations

FENO: Fraction of exhaled nitric oxide; IT: Immunotherapy; ACT: Asthma control test; ARCS: Allergic rhinoconjunctivitis symptoms; BMI: Body mass index.

## Competing interests

All authors declare that they have no competing financial interests. This study was funded by the Sharpe-Strumia Research Foundation of the Bryn Mawr Hospital, Bryn Mawr, PA, USA (SSRF 2007–05, 2008–08, 2010–09).

## Authors’ contributions

MC participated in the study design, acquisition, interpretation and analysis of the data, in drafting and revising the manuscript. BW participated in the analysis, interpretation of the data, and in revising the manuscript. RAP participated in the interpretation of the data, and in revising the manuscript. ASR participated in the study design, acquisition, interpretation of the data, and in revising the manuscript. All authors approved the final version of this manuscript.
